# First Clinical Evidence Linking Smoking to Increased Postoperative Macular and Retinal Nerve Fiber Layer Thickness in Cataract Surgery Patients: A Prospective Cohort Study

**DOI:** 10.3390/jcm14124131

**Published:** 2025-06-11

**Authors:** Darko Batistic, Sandro Glumac, Jozefina Josipa Dukic, Filip Rada, Josip Vrdoljak, Jaksa Batistic, Braco Boskovic, Maja Mizdrak, Ante Kreso

**Affiliations:** 1 Department of Ophthalmology, University Hospital of Split, 21000 Split, Croatia; batisticdarko@gmail.com (D.B.); josipa.duk@gmail.com (J.J.D.); filiprada22@gmail.com (F.R.); akreso345@gmail.com (A.K.); 2 Department of Anesthesiology and Intensive Care, University Hospital of Split, 21000 Split, Croatia; 3 Department of Pathophysiology, School of Medicine, University of Split, 21000 Split, Croatia; j.vrdoljak9@gmail.com (J.V.); maja.mizdrak@mefst.hr (M.M.); 4 Department of Urology, University Hospital of Split, 21000 Split, Croatia; jbatistic@gmail.com; 5 Department of Otorhinolaryngology, University Hospital of Split, 21000 Split, Croatia; bboskovic01@gmail.com; 6 Department of Nephrology and Hemodialysis, University Hospital of Split, 21000 Split, Croatia

**Keywords:** cataract surgery, smoking, macular edema, optical coherence tomography, phacoemulsification, cube volume, cube average thickness, postoperative retinal changes

## Abstract

**Background**: Postoperative macular edema may limit visual recovery following cataract surgery. Although smoking is recognized as a risk factor for ocular inflammation, its impact on early postoperative macular morphology following cataract surgery has not been investigated. **Methods**: This prospective cohort study enrolled 88 elderly patients undergoing elective cataract surgery in a single university teaching hospital. The patients were divided into long-term smokers and lifelong non-smokers. Spectral-domain optical coherence tomography (OCT) was used to assess the central subfoveal thickness (CST), cube volume (CV), cube average thickness (CAT), retinal nerve fiber layer (RNFL), and cup-to-disk ratio (CDR) preoperatively and on the 1st, 7th, and 14th postoperative days (PODs). The phacoemulsification time and cumulative dissipated energy were recorded. Linear mixed-effects models were used to assess group-by-time interactions, and multivariable regression, adjusted for baseline covariates, was employed for analyses. **Results:** Eighty patients were included in the final analysis. Smokers had significantly thinner baseline CST than non-smokers. Both groups showed early postoperative CST increases, but only smokers exhibited sustained and significantly greater increases in CV and CAT on POD 14 (CV Δ +0.30 mm^3^ vs. +0.04 mm^3^; *p* = 0.026; CAT Δ +6.5 µm vs. +1.2 µm; *p* = 0.037). The RNFL and CDR changes did not differ significantly at earlier timepoints. However, smokers showed a notably greater RNFL thickening on POD 14 (Δ +4.2 µm; *p* = 0.001). Smoking status remained the strongest independent predictor of these changes (*p* < 0.001), while phacoemulsification parameters showed no significant interaction effects. **Conclusions**: Cigarette consumption independently predicts pronounced postoperative macular and RNFL thickening after uncomplicated elective cataract surgery. These transient structural changes could complicate early glaucoma assessment and should be considered when interpreting postoperative OCT findings in smokers.

## 1. Introduction

Cataract surgery is a common and very helpful surgical procedure, with over 28 million operations performed annually [1]. Compared to previously used methods, current cataract surgical techniques, particularly phacoemulsification, have significantly improved visual outcomes and decreased postoperative complication rates [2].

Cigarette smoking is one of the major public health threats facing the world, killing over 7.5 million people each year worldwide [3]. It has been widely recognized as a risk factor for cataract development and progression [4]. Furthermore, numerous studies have emphasized the association of smoking with the occurrence of ocular inflammation [5,6]. However, in addition to accelerating the appearance of cataracts, smoking changes the retinal tissue thickness as well, specifically by reducing the retinal nerve fiber layer (RNFL) [7].

Several studies have shown increased macular thickness following uncomplicated cataract surgery, which is attributed to the inflammatory response and altered fluid dynamics that occur during the early postoperative period [8,9]. Nevertheless, the potential exaggeration of this effect in smokers is still poorly understood, requiring further investigation. Smoking-induced chronic inflammation and oxidative stress [10] may exacerbate the postoperative inflammatory response, potentially leading to more pronounced macular thickening. Optical coherence tomography (OCT) angiography has recently confirmed smoking-related microvascular compromise, as chronic smokers show reduced macular vessel density and an enlarged foveal avascular zone, and a recent systematic review likewise found that smoking lowers the choroidal vascularity index without appreciably altering the mean retinal thickness [11,12]. While previous studies have focused more on the general characteristics of patients and their other ocular diseases [13,14], the specific impact of smoking on changes in macular thickness after cataract surgery has not yet been properly investigated.

OCT is a non-invasive imaging technique that uses reflected light to create images of the posterior segment of the eye. It has emerged as a valuable diagnostic tool for analyzing the optic nerve head and quantifying retinal changes [15].

Therefore, the current study sought to explore the repercussions of cigarette consumption on postoperative macular thickness, RNFL thickness, and choroidal thickness, as measured by OCT, in patients undergoing elective cataract surgery. We hypothesized that smoking status represents an additional independent risk factor for macular changes following cataract extraction in smokers compared to non-smokers. Thus, the results of this research may demonstrate a need for preoperative counseling and postoperative management strategies for patients who have a history of smoking and are planning to undergo cataract surgery.

## 2. Materials and Methods

### 2.1. Study Design

This prospective cohort study was performed at the University Hospital of Split, Croatia, between July 2024 and March 2025. This paper adheres to the applicable STROBE guidelines, and the research was conducted in accordance with the principles of the Declaration of Helsinki. Ethical approval for this study (Ethical Committee No. 2181–147/01/06/LJ.Z.-23-02) was provided by the Ethical Committee of the University Hospital of Split (Chairperson Prof Lj. Znaor) on 21 July 2022. All patients provided written informed consent.

### 2.2. Study Participants

This study enrolled patients who were scheduled for elective cataract surgery with local anesthesia; patients were older than 60 years with a smoking history longer than 10 years and consumed at least 10 cigarettes per day [16]. Exclusion criteria were any other ocular diseases that may impair macular thickness, diabetes mellitus type 1 or 2, uncontrolled hypertension (untreated hypertension or poorly controlled hypertension with blood pressure above 150/90 mmHg), chronic obstructive pulmonary disease, liver or renal illness, a rheumatic or endocrine disorder, steroid treatment for a prolonged period, alcohol consumption (>20 g per day or >150 g per week), and body mass index (BMI) over 30 kg/m^2^. If blood pressure measured non-invasively on the upper arm was above 160/95 mmHg or pulse oximetry on the index finger registered peripheral capillary oxygen saturation lower than 94% upon admission to the operating theater, the patient was excluded from the study [17]. Furthermore, a patient was excluded if any complication arose intraoperatively. Finally, a patient was excluded from the study if the signal strength observed in the OCT scan was weaker than 5/10 or if the patient’s smoking pattern changed during the early postoperative period.

### 2.3. OCT and Other Measurements

Detailed medical history was collected, and a complete physical examination was conducted for each patient prior to the surgery. Special attention was paid to collecting precise data regarding the duration of smoking and the number of cigarettes consumed daily. Furthermore, preoperative measurements that were taken one day before surgery consisted of determining the degree of cataract using the LOCS 3 classification, performing OCT analysis (Zeiss Cirrus HD-OCT 400, Carl Zeiss Meditec Inc., Jena, Germany) of the macula and optic nerve head, and conducting OCT angiography of the macula. Phacoemulsification time and cumulative dissipated energy (CDE) were recorded at the end of each surgery as measures correlating with induced surgical inflammation and thermal and mechanical stress. [18] OCT analyses in the patients were repeated on the 1st, 7th, and 14th postoperative days (PODs). The same investigator (AK) performed all the OCT measurements to ensure consistency during data collection.

### 2.4. Surgery and Anesthesia

Cataract surgery was performed as an outpatient procedure and involved the administration of a local anesthetic (three drops of oxybuprocaine were applied onto the ocular surface three times during a time period of 20 min, just before the surgery), in addition to systemic sedation (5 mg diazepam orally administered by a nurse, 30 min before the procedure). All surgical procedures were carried out using the same surgical technique, i.e., stop-and-chop technique, considering that it represents an excellent all-around phacoemulsification technique that can efficiently address a broad range of cataracts—from moderate to very dense. The technique began with the creation of a central groove. Next, the chopper was inserted into the groove, and a horizontal movement was performed to crack the nucleus in half. The cracking procedure was then stopped, the ultrasound tip was impaled into one hemi-nucleus, and a vertical chop was then performed if necessary. After completing the stop-and-chop technique and removing the cataract, an intraocular lens (AcrySof^®^ IQ aspheric IOL, Alcon Laboratories Inc., Fort Worth, TX, USA) was implanted into the capsular bag. To further minimize the variability, all surgeries were performed by a single surgeon (DB).

### 2.5. Primary Endpoint

The primary endpoint in the current study was the difference in macular thickness and RNFL thickness, measured by OCT, between smokers and non-smokers after elective cataract surgery. Postoperative day 14 was selected as the primary endpoint because it coincides with the first routine review in our in-house cataract protocol and encompasses the period during which macular thickness after uncomplicated phacoemulsification is known to increase [13,19,20].

### 2.6. Secondary Outcome Measure

The secondary outcome measure included the effect of phacoemulsification time and CDE on the degree of postoperative macular and RNFL thickening in smokers in comparison with non-smokers.

### 2.7. Statistical Analysis

Data analysis was performed using IBM SPSS Statistics, version 28.0 (IBM Corp., Armonk, NY, USA). Descriptive statistics were used to summarize baseline characteristics. Continuous variables are presented as means ± standard deviations or medians with interquartile ranges, depending on the data distribution. Categorical variables are presented as counts and percentages. Group comparisons between smokers and non-smokers were performed using independent-sample *t*-tests for normally distributed continuous variables, Mann–Whitney U tests for skewed data, and chi-square or Fisher’s exact tests for categorical variables.

Longitudinal outcomes (central subfoveal thickness (CST), cube volume (CV), cube average thickness (CAT), RNFL, and cup-to-disk ratio (CDR)) were assessed preoperatively and at three postoperative timepoints. Between-group differences at each timepoint were analyzed using appropriate univariate tests. To evaluate temporal patterns and group-by-time interactions, linear mixed-effects models were constructed for each OCT parameter with fixed effects for group, time, and their interaction and a random intercept for each subject. Interaction significance was tested via likelihood-ratio tests by comparing full models to reduced models without the interaction term. To explore predictors of structural changes, we used two complementary approaches. First, multivariable linear regressions with step-wise backward elimination were applied to each postoperative change (Δ) as the dependent variable. Second, to capture the entire time course while accounting for the non-independence of repeated measures within the same eye, we fitted linear mixed-effects models with a random intercept per eye. In both approaches, the fixed covariates were baseline OCT value, age, smoking status, phacoemulsification time, cumulative dissipated energy (CDE), and axial length, thus controlling for the main surgical and ocular factors known to influence early macular thickening. Potential interaction effects between operative parameters and smoking status were examined by adding the relevant interaction terms to stratified models. Model coefficients were estimated with Satterthwaite-adjusted degrees of freedom, and statistical significance was set at two-sided *p* < 0.05.

The sample size was calculated a priori to detect a minimum between-group difference of 8 µm in CST with 80% power and a two-sided α of 0.05, assuming a standard deviation of 12 µm. This yielded a required sample of 36 eyes per group. Considering an estimated attrition rate of about 10%, the final sample size was increased to a total of 88 patients (i.e., 44 patients per group). IBM SPSS Statistics, version 28.0, was used to calculate the required sample size.

## 3. Results

### 3.1. Study Population

Between July 2024 and March 2025, 163 patients were screened, and 102 of these patients matched the selection criteria. Among the eligible patients, 88 patients (44 patients in the smoking group and 44 patients in the non-smoking group) provided written informed consent and were enrolled in the study. Five patients had preoperatively elevated blood pressure levels, one patient developed an intraoperative complication, and in two patients, the signal strength in OCT examination scans was poor. Ultimately, we analyzed the data on 80 patients (40 patients in the smoking group and 40 patients in the non-smoking group). The baseline demographic, clinical, and OCT characteristics of the patients are presented in Table 1. The groups were well balanced for sex distribution, BMI, intraoperative CDE, and all other baseline parameters, apart from age and CST. Smokers were, on average, 3.8 years younger than non-smokers (70.1 ± 5.5 y vs. 73.8 ± 4.8 y; *p* = 0.002) (Table 1). In addition, on average, smokers had a lower CST baseline than non-smokers (253.34 ± 15.10 vs. 262.24 ± 22.47, *p* = 0.041) (Table 1).

### 3.2. Cross-Sectional Comparisons at Each Timepoint

**Figure 1:**
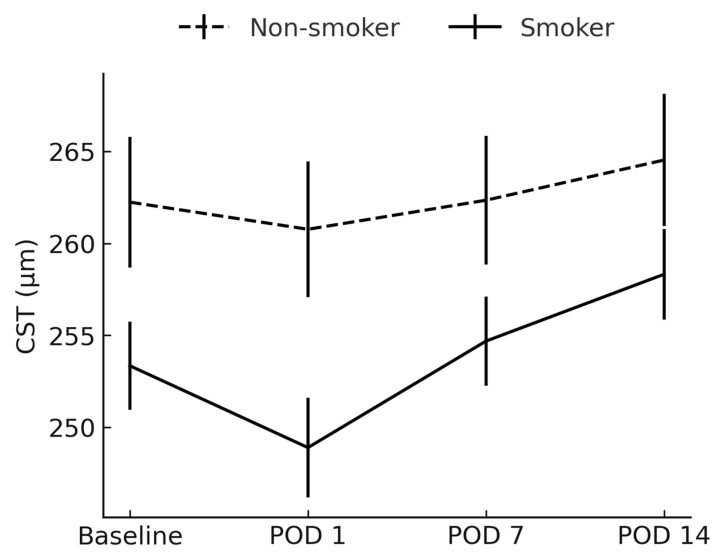
Comparison of central subfoveal thickness between smokers and non-smokers. CST = central subfoveal thickness; POD = postoperative day.

CST was significantly lower in smokers preoperatively (mean difference −9 µm, *p* = 0.041) and on POD 1 (mean difference −8 µm, *p* = 0.044), but not at later follow-ups (Figure 1).

**Figure 2:**
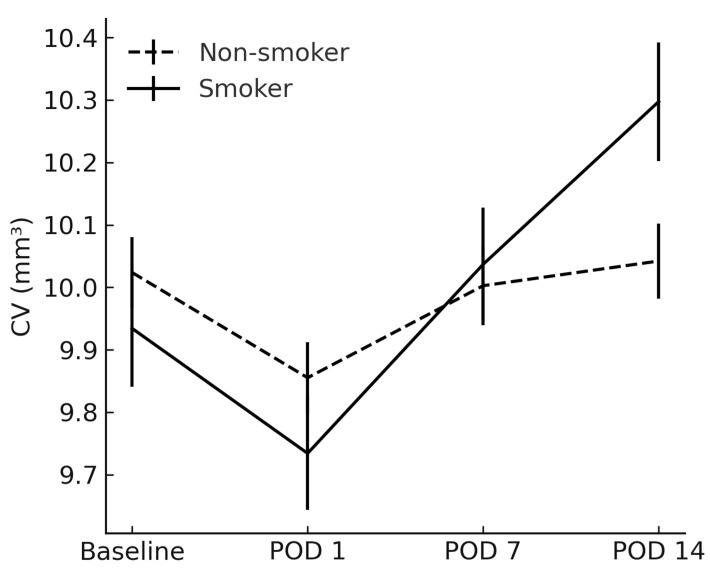
Comparison of cube volume between smokers and non-smokers. CV = cube volume; POD = postoperative day.

CV had become significantly higher in smokers by POD 14 (10.30 ± 0.60 mm^3^ vs. 10.04 ± 0.38 mm^3^; *p* = 0.026) (Figure 2). Post hoc inspection of mean CST and CV trajectories revealed that the inter-group difference first reached statistical significance between POD 7 and POD 14, indicating a ≈1-week “window of vulnerability” during which smoking-related inflammatory amplification is most pronounced.

**Figure 3:**
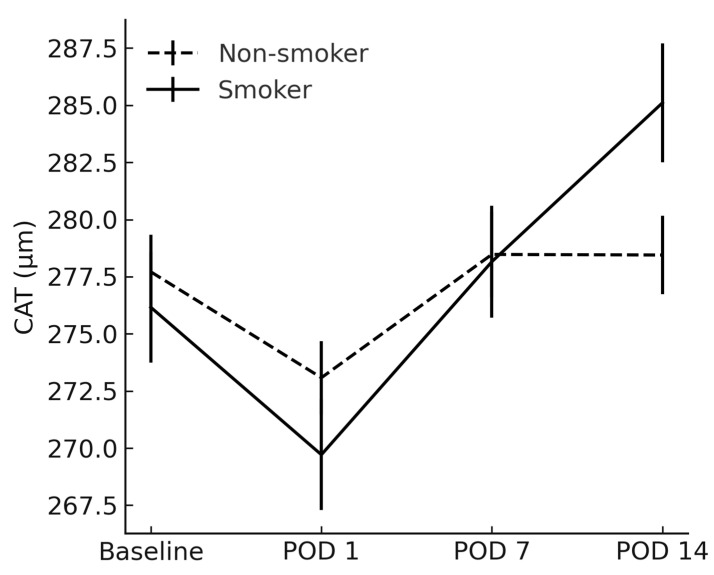
Comparison of cube average thickness between smokers and non-smokers. CAT = cube average thickness; POD = postoperative day.

Similarly, CAT in smokers exceeded that in non-smokers on POD 14 (285.1 ± 16.5 µm vs. 278.5 ± 10.9 µm; *p* = 0.037) (Figure 3).

**Figure 4:**
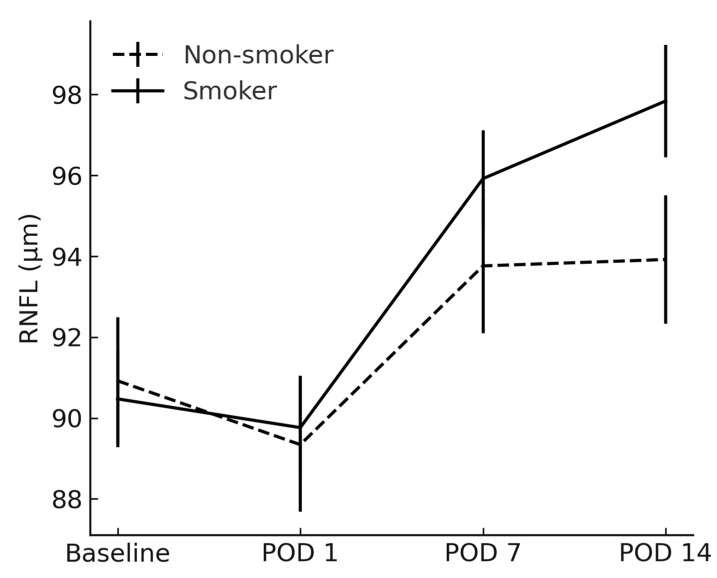
Comparison of retinal nerve fiber layer between smokers and non-smokers. RNFL = retinal nerve fiber layer; POD = postoperative day.

**Figure 5:**
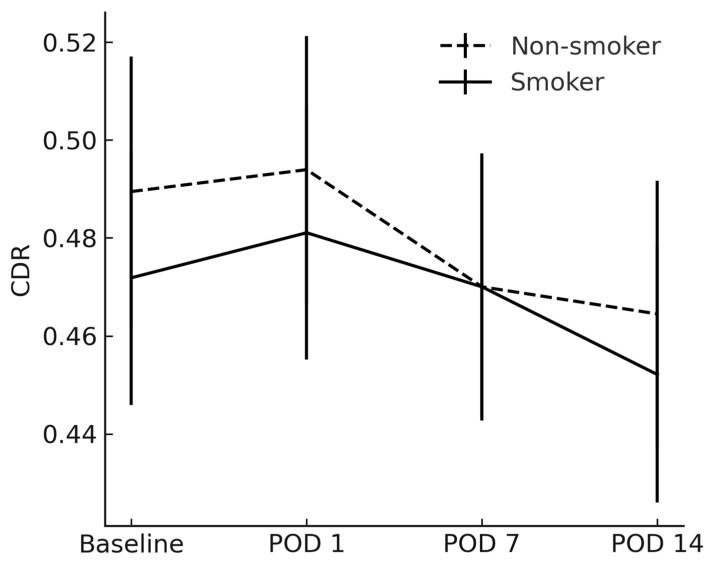
Average cup-to-disk ratio between smokers and non-smokers. CDR = cup-to-disk ratio; POD = postoperative day.

The RNFL and CDR showed no between-group differences at any timepoint (*p* > 0.05) (Figure 4 and Figure 5).

No significant inter-group differences were observed on POD 1 or POD 7; the earliest detectable divergence occurred on POD 14, indicating that any smoking-related exacerbation of macular thickening manifested within the first two postoperative weeks.

### 3.3. Mixed-Effects Longitudinal Analysis

To formally test whether trajectories differed over time, each outcome was modeled with a linear mixed-effects model. A likelihood-ratio (LR) test compared the full model to one without the interaction term.

Only CV and CAT exhibited a statistically significant interaction, confirming that smokers follow a different postoperative course compared with non-smokers (Table 2).

### 3.4. Association of Phacoemulsification Time with Postoperative Changes

After adjusting for the baseline value, age, and smoking status, the multivariate linear regression analysis results showed that phacoemulsification time was not an independent predictor of change in any OCT parameter on POD 14 (all *p* > 0.10). Point estimates suggested modest positive slopes for CAT (+0.05 µm per minute) and CST (+0.02 µm per minute), but confidence intervals crossed zero (Table 3). Age was retained as a covariate in all models to adjust for the small but significant baseline difference between groups.

In contrast, smoking status remained a strong independent correlate of postoperative change. Compared with non-smokers, smokers showed a +0.30 mm^3^ greater increase in CV (*p* < 0.001), a +6.5 µm greater rise in CAT (*p* < 0.001), +4.2 µm greater RNFL thickening (*p* = 0.001), and +2.6 µm higher CST rebound (*p* = 0.022). These findings indicate that the longer intraoperative phacoemulsification time itself has minimal explanatory value once age and baseline anatomy are considered, whereas smoking status consistently predicts larger structural changes during the first two postoperative weeks.

### 3.5. Multivariable Regression Across Sequential Time Intervals

To explore differences based on smoking status, we fitted a multivariable model. Multivariable models with interaction terms (Phaco_minutes × Group and CDE × Group) showed no statistically significant effect modification for any OCT outcome across the three postoperative intervals (all interactions *p* ≥ 0.08; Table 4). In other words, the incremental impact of phacoemulsification time and cumulative delivered energy on retinal changes was comparable in smokers and non-smokers. The greater macular thickening observed in smokers is therefore unlikely to be mediated by increased susceptibility to operative energy but rather reflects intrinsic smoking-related tissue responses.

## 4. Discussion

The current prospective cohort study on 80 cataract surgery patients showed a significant difference in OCT analysis between smokers and non-smokers. To our knowledge, this is the first study to reveal that cigarette consumption drives postoperative macular alterations, where smokers experience notably larger increases in overall macular volume and average thickness. Although both smokers and non-smokers experienced early swelling at the very center of the macula, only smokers developed a sustained and greater rise in these broader volume and thickness measurements two weeks after phacoemulsification. Although our observations ended at 2 weeks, a layer-by-layer analysis demonstrated that inner retinal layers continued to thicken after POD 14, with thickening peaking by 1 month and regressing by 3 months postoperatively [13,19,20]. In contrast, changes in the RNFL and the optic nerve head (such as CDR) were similar regardless of smoking status, except at POD 14. Statistical modeling confirmed that the combination of smoking and the time period following surgery drove the macular volume and thickness increases, and further analysis showed that smoking was a more powerful predictor of these effects than any differences in surgical energy or duration. Comparatively, eyes undergoing femtosecond laser-assisted surgery showed less inner retinal thickening at 2 weeks and beyond, indicating a dose–dependent response to ultrasound energy [21]. Nevertheless, these early structural differences should be interpreted cautiously until long-term functional correlates are confirmed.

Our findings extend previous evidence on postoperative macular changes following phacoemulsification while identifying a distinct role of smoking. Recent studies, such as those by Anastasilakis et al. [8], Ilveskoski et al. [9], and Mackenbrock et al. [22], reported that longer phacoemulsification time and higher CDE are associated with greater postoperative macular thickening. However, none of these studies isolated the effect of smoking as an independent contributor.

Most earlier investigations either focused on diabetic or uveitis populations [13,14] or evaluated chronic smoking effects on baseline retinal anatomy [7,23,24], without exploring postoperative dynamics. On the other hand, we demonstrated that smoking history alone predicts significantly greater increases in macular CV and CAT within the early postoperative period, independently of surgical parameters. This can be clinically relevant because it suggests that the observed macular edema in smokers is not merely a byproduct of intraoperative stress, but rather a reflection of smoking-induced chronic tissue susceptibility, likely mediated by microvascular dysfunction, oxidative stress, and impaired blood–retinal barrier integrity. Furthermore, while previous studies confirmed that smoking reduces baseline retinal thickness [25,26], our data show that this thinner starting point does not mitigate but rather worsens exaggerated postoperative thickening. By enrolling a tightly defined cohort and using standardized surgical and imaging protocols, this study adds new evidence that could help explain individual variation in macular recovery after cataract surgery.

Macular edema in ocular diseases, characterized by intraocular inflammation, such as diabetic retinopathy and uveitis, arises from the increased vascular permeability of parafoveal capillaries, manifesting as elevations in CAT and CV [27,28]. The vitreous body acts as a reservoir for inflammatory mediators (such as interleukins and cytokines), which promote the breakdown of the blood–retinal barrier. Moreover, the choroid exhibits the highest blood flow per unit volume of any human tissue, rendering the macula particularly susceptible to hemodynamic and inflammatory insults [29]. Free radicals from smoking concentrate preferentially in this region, exacerbating oxidative damage [30,31]. These combined mechanisms likely underlie the augmented late postoperative thickening seen in smokers.

Anatomically, the greater increase in macular thickness versus RNFL thickness reflects the dense parafoveal capillary network surrounding the foveal avascular zone (FAZ), where CST is measured and where exudation is minimal [32]. Indeed, CST exhibited the smallest postoperative rise in both groups, consistent with limited leakage in the FAZ. Finally, it has already been established, and our data further confirm, that baseline CST is thinner in smokers than in non-smokers, indicating central neuro-sensory retinal thinning in this population [25].

Although there was no statistically significant difference in RNFL thickness during the initial postoperative assessments, smoking status was clearly associated with a more pronounced increase in RNFL thickness two weeks following surgery. This finding could be clinically important, as it suggests that RNFL measurements in smokers may be temporarily elevated in the early postoperative period. In the context of glaucoma management, such transient thickening could lead to the misinterpretation of structural stability or progression, given that RNFL thinning is a key parameter in monitoring disease [33]. Because of these changes in RNFL thickness, clinicians should interpret early postoperative OCT scans of the optic nerve with caution in smokers.

Since this study was limited to a 14-day postoperative period, the evolution of these changes over time and their potential long-term clinical significance for glaucoma remain unclear. Extended follow-up is needed to determine how long the RNFL thickening observed in smokers persists.

Cataract surgery itself provokes a pro-inflammatory response through prostaglandin release from the iris (i.e., “Irvine–Gass” syndrome), which contributes to early macular swelling [34,35]. Intriguingly, we observed a greater reduction in CV among smokers compared to non-smokers on POD 1, a finding that may reflect reduced choroidal perfusion and blunted acute inflammatory recruitment due to smoking-induced vascular dysfunction. However, it may be that elevated concentrations of vitreous inflammatory mediators in smokers then drive a delayed and progressively increasing edema over subsequent days. Smoking also disrupts both choroidal and retinal circulation and may alter capillary wall properties, potentially delaying vascular fenestration and early fluid accumulation, though the precise mechanisms remain ambiguous [12,36].

By POD 14, all smokers exhibited significantly higher CV and CAT compared with non-smokers, yet the duration of this pro-inflammatory thickening remains unknown. Longitudinal studies are required to determine how long smoking-related macular edema persists and whether it portends poorer visual or patient-reported outcomes. In line with previous studies showing that higher cumulative dissipated energy during phacoemulsification is related to more pronounced postoperative macular thickening, we also observed a modest but significant correlation between CDE and increases in CV and CAT [8,9]. Clinically, these findings highlight the need for enhanced preoperative counseling and closer OCT monitoring in smokers, as well as the potential benefit of energy-sparing phacoemulsification techniques (e.g., chopping strategies, pulse-mode ultrasound, increased irrigation) to mitigate intraoperative tissue stress.

This study has several limitations. First, its single-center design and relatively small sample size (n = 80) may limit external validity. Second, smoking exposure was self-reported and not biochemically verified, and precise dose–response effects could therefore not be assessed. Third, follow-up was confined to 14 days, and functional outcomes, such as best-corrected visual acuity or contrast sensitivity, were not collected, so the durability and clinical relevance of the observed structural changes remain uncertain. Finally, systemic inflammatory markers were not measured, and residual age-related or surgical confounders cannot be completely ruled out despite multivariable adjustment. Future studies with longer follow-ups, more objective smoking verification, and functional endpoints should also evaluate whether intensified peri-operative anti-inflammatory prophylaxis confers particular benefits to smokers.

## 5. Conclusions

Within the first 14 days after uncomplicated elective cataract surgery, current smokers exhibited small but statistically significant increases in central macular volume and cube average thickness compared with non-smokers. Although the absolute differences were modest and no functional measures were collected, these findings suggest that smoking may amplify the early inflammatory response to surgery. Given the limited follow-up and lack of visual outcomes, the clinical implications remain uncertain, and routine OCT interpretation in smokers should therefore be approached with caution until longer-term data are available.

## Figures and Tables

**Table 1:** Demographic, clinical, and OCT characteristics.

Characteristics	Smokers (n = 40)	Non-Smokers (n = 40)	*p*-Value
Age (yrs)	70.05 ± 5.54	73.84 ± 4.77	0.002 *
Male sex	18 (47.4%)	18 (47.4%)	1.00 ‡
Female sex	20 (52.6%)	20 (52.6%)	1.00 ‡
BMI (kg/m^2^)	23.55 [22.12–24.85]	24.65 [22.18–28.32]	0.189 †
CDE	9.05 [6.80–14.56]	8.21 [6.32–11.13]	0.170 †
CST 0 (µm)	253.34 ± 15.10	262.24 ± 22.47	0.041 *
CV 0 (mm^3^)	9.93 ± 0.59	10.02 ± 0.36	0.415 *
CAT 0 (µm)	276.16 ± 15.37	277.71 ± 10.24	0.597 *
CST 1 (µm)	249.50 [242–259]	257.50 [245–273]	0.044 †
CV 1 (mm^3^)	9.73 ± 0.57	9.86 ± 0.36	0.262 *
CAT 1 (µm)	269.71 ± 15.34	273.08 ± 10.17	0.251 *
CST 2 (µm)	254.68 ± 15.31	262.34 ± 22.09	0.076 *
CV 2 (mm^3^)	10.04 ± 0.58	10.00 ± 0.40	0.759 *
CAT 2 (µm)	278.16 ± 15.45	278.47 ± 11.80	0.918 *
CST 3 (µm)	258.32 ± 15.60	264.53 ± 22.75	0.159 *
CV 3 (mm^3^)	10.30 ± 0.60	10.04 ± 0.38	0.026 *
CAT 3 (µm)	285.11 ± 16.50	278.45 ± 10.87	0.037 *
RNFL 0 (µm)	90.47 ± 7.60	90.92 ± 9.91	0.821 *
Avg. CDR 0	0.53 [0.46–0.58]	0.52 [0.46–0.60]	0.596 †
RNFL 1 (µm)	89.76 ± 8.07	89.34 ± 10.51	0.841 *
Avg. CDR 1	0.54 [0.48–0.59]	0.53 [0.45–0.61]	0.840 †
RNFL 2 (µm)	95.92 ± 7.59	93.76 ± 10.51	0.296 *
Avg. CDR 2	0.52 [0.45–0.59]	0.52 [0.42–0.58]	0.908 †
RNFL 3 (µm)	97.84 ± 8.80	93.92 ± 10.06	0.067 *
Avg. CDR 3	0.49 [0.43–0.57]	0.51 [0.42–0.57]	0.795 †

OCT = optical coherence tomography; BMI = body mass index; CDE = cumulative dissipated energy; 0 = baseline; 1 = postoperative day 1; 2 = postoperative day 7; 3 = postoperative day 14; CST = central subfoveal thickness; CV = cube volume; CAT = cube average thickness; RNFL = retinal nerve fiber layer; Avg. CDR = average cup-to-disk ratio. * = *t*-test for normal distribution; † = Mann–Whitney U-test (non-normal continuous data); ‡ = χ^2^ test (categorical data).

**Table 2:** Effects of time course and cigarette consumption on OCT parameters.

Variable	LR χ^2^ (df = 3)	*p* (Group × Time)
CST (µm)	1.8	0.618
CV (mm^3^)	13.0	0.0046
CAT (µm)	9.3	0.0100
RNFL (µm)	4.3	0.235
Avg. CDR	0.1	0.978

OCT = optical coherence tomography; CST = central subfoveal thickness; CV = cube volume; CAT = cube average thickness; RNFL = retinal nerve fiber layer; Avg. CDR = average cup-to-disk ratio.

**Table 3:** Factors correlating with changes in OCT parameters on POD 14: phacoemulsification time and smoking.

**Outcome (Δ)**	**β Phaco (95% CI)**	***p* Phaco**	**β Smoker vs. NS**	***p* Smoker**	**Adj. R^2^**
CST (µm)	+0.016 µm/min (−0.019 to +0.052)	0.362	+2.58 µm	0.022	0.085
CV (mm^3^)	+0.0004 mm^3^/min (−0.0016 to +0.0024)	0.685	+0.30 mm^3^	<0.001	0.389
CAT (µm)	+0.045 µm/min (−0.010 to +0.100)	0.105	+6.53 µm	<0.001	0.327
RNFL (µm)	−0.005 µm/min (−0.048 to +0.038)	0.812	+4.22 µm	0.001	0.175
Avg. CDR	−0.0000/min (−0.0002 to +0.0002)	0.901	+0.005	0.274	−0.020

Model: Δ POD 14 = β_0_ + β_1_ Phaco_minutes + β_2_ Smoking group + β_3_ Age + β_4_ Baseline value. OCT = optical coherence tomography; POD = postoperative day; NS = non-smoker group; CST = central subfoveal thickness; CV = cube volume; CAT = cube average thickness; RNFL = retinal nerve fiber layer; Avg. CDR = average cup-to-disk ratio.

**Table 4:** Slopes for operative parameters stratified by smoking status.

Outcome Δ	Interval	Phaco β NS	Phaco β S	*p* Phaco	CDE β NS	CDE β S	*p* CDE
CST (µm)	0→1 d	+0.1036 µm/min	−0.0543 µm/min	0.103	+0.1227 µm/pc-s	+0.0921 µm/pc-s	0.947
CST (µm)	1→7 d	−0.0506	+0.1275	0.080	−0.0730	−0.1952	0.799
CST (µm)	7→14 d	−0.0292	−0.0360	0.853	+0.0304	−0.0252	0.754
CV (mm^3^)	0→1 d	+0.0008 mm^3^/min	−0.0006 mm^3^/min	0.469	−0.0011 mm^3^/pc-s	+0.0056 mm^3^/pc-s	0.460
CV (mm^3^)	1→7 d	−0.0004	+0.0011	0.464	+0.0002	−0.0042	0.663
CV (mm^3^)	7→14 d	−0.0018	+0.0009	0.127	+0.0063	−0.0009	0.385
CAT (µm)	0→1 d	+0.0282 µm/min	+0.0141 µm/min	0.809	−0.0603 µm/pc-s	−0.0777 µm/pc-s	0.950
CAT (µm)	1→7 d	−0.0585	+0.0738	0.022	+0.5364	−0.2277	0.006
CAT (µm)	7→14 d	−0.0170	+0.0653	0.080	−0.2785	−0.1488	0.566
RNFL (µm)	0→1 d	+0.0057 µm/min	+0.0421 µm/min	0.455	+0.2889 µm/pc-s	−0.0245 µm/pc-s	0.181
RNFL (µm)	1→7 d	−0.0079	−0.0387	0.428	+0.0172	+0.1448	0.493
RNFL (µm)	7→14 d	−0.0014	+0.0162	0.552	−0.1373	−0.0484	0.532
Avg. CDR	0→1 d	−0.0001	+0.0001	0.163	−0.0004	−0.0002	0.781
Avg. CDR	1→7 d	+0.0001	0	0.584	−0.0009	+0.0002	0.322
Avg. CDR	7→14 d	−0.0001	0	0.872	+0.0005	−0.0006	0.239

Positive β indicates a greater increase in the outcome per unit of the predictor; pc-s = percent-seconds (CDE units). Interaction p tests whether slopes differ between smokers and non-smokers. NS = non-smokers; S = smokers; CDE = cumulative dissipated energy; d = day; CST = central subfoveal thickness; CV = cube volume; CAT = cube average thickness; RNFL = retinal nerve fiber layer; Avg. CDR = average cup-to-disk ratio.

## Data Availability

The datasets generated and analyzed during the current study are not publicly available due to patient privacy considerations, but are available from the corresponding author upon reasonable request.

## Abbreviations

The following abbreviations are used in this manuscript:

AMD	age-related macular degeneration
BMI	body mass index
CAT	cube average thickness
CDR	cup-to-disk ratio
CDE	cumulative dissipated energy
CI	confidence interval
CST	central subfoveal thickness
CV	cube volume
FAZ	foveal avascular zone
GBD	Global Burden of Disease
IOL	intraocular lens
LOCS 3	Lens Opacities Classification System III
LR	likelihood ratio (test)
NSAID	non-steroidal anti-inflammatory drug
NS	non-smokers (group identifier)
OCT	optical coherence tomography
pc-s	percent-seconds (unit used for CDE)
POD	postoperative day
RNFL	retinal nerve fiber layer
S	smokers (group identifier)
SPSS	(IBM) Statistical Package for the Social Sciences
STROBE	Strengthening the Reporting of Observational Studies in Epidemiology
